# Human milk and total milk intakes of mixed fed infants: a cross-sectional study of infants aged 7–10 months

**DOI:** 10.1007/s00394-026-03973-1

**Published:** 2026-04-28

**Authors:** Lisa Daniels, Anne-Louise M. Heath, Rachael W. Taylor, Bailey Bruckner, Aly Diana, Isabella Zinzan-Dickie, Neve H. McLean, Alice M. Cox, Emily A. Jones, Ioanna Katiforis, Kimberley J. Brown, Madeleine Rowan, Maria Casale, Rosario Jupiterwala, Michelle Harper, Cathryn A. Conlon, Kathryn L. Beck, Lisa Te Morenga, Pamela R. von Hurst, Jillian J. Haszard

**Affiliations:** 1 Department of Human Nutrition, University of Otago, Dunedin, 9054 New Zealand; 2 Department of Medicine, University of Otago, Dunedin, New Zealand; 3 Department of Public Health, Faculty of Medicine, Universitas Padjadjaran, Bandung, Indonesia; 4 School of Sport, Exercise and Nutrition, Massey University, Auckland, New Zealand; 5 Research Centre for Hauora and Health, Massey University, Wellington, New Zealand; 6 Haszard Biostatistics, Otago, New Zealand

**Keywords:** Human milk intake, Breast milk intake, Stable isotope, Infant formula intake, Infant

## Abstract

**Background/objectives:**

There is a paucity of data on the volume of human milk consumed by infants, particularly in later infancy (> 6 months). The objective was to estimate human milk intakes and total infant milk intakes (i.e. including infant formula), and describe associated correlates in New Zealand (NZ) infants aged 7–10 months.

**Subjects/methods:**

First Foods NZ (FFNZ) was a cross-sectional study that investigated infant feeding in NZ (July 2020-February 2022). Human milk intakes were obtained using the dose-to-mother stable isotope technique in a subset of breastfeeding mother-infant dyads. Infant formula intake was determined from 24-h recalls. Sociodemographic information was collected through questionnaires, and anthropometric measurements made. Total infant milk intake referred to human milk and infant formula.

**Results:**

The breastfeeding sub-study comprised 157 mother-infant dyads whose infant milk was fully or partially human milk, and 217 non-breastfeeding dyads. All infants consumed solid foods. The mean (SD) human milk intake was 785 (264) g/day, higher in those consuming only human milk and no infant formula (828 g/day), and decreasing with age. Total infant milk intakes were around 820 g/day, regardless of milk type. Factors associated with both human milk and total infant milk intakes were infant age, BMI z-score, and maternal education. Maternal BMI and demand feeding were also related to human milk intakes. There was no difference in milk intake by season.

**Conclusions:**

This study provides valuable insight into human milk intakes during late infancy in NZ infants (7–10 months), improving our ability to accurately assess nutritional intakes and nutrient adequacy of infants.

**Supplementary Information:**

The online version contains supplementary material available at 10.1007/s00394-026-03973-1.

## Introduction

Infant milk, whether human milk or infant formula, forms a major part of the diet during infancy [1]. However, accurate measures of how much human milk infants are receiving, particularly in later infancy (> 6 months of age) when solid foods and other fluids are introduced, remains challenging but important to determine, because accurate estimations affect the ability to understand true nutrient intakes and adequacy.

Whereas quantifying infant formula intake is straightforward, accurately estimating human milk intake is difficult and time-consuming. A “one size fits all” method is most commonly used to estimate human milk intake, where each infant of the same age is presumed to consume an identical volume (e.g., 600 mL/day for an infant aged 7–12 months) [2, 3]. However, this approach fails to account for the inherent variability in human milk intakes which occurs even in exclusively breastfed infants [4]. An alternative method, test-weighing, involves weighing the infant before and after each feed. This approach is time-consuming, can potentially disturb normal feeding patterns, and may overlook minor feeding instances, impacting the accurate estimation of human milk intakes [5].

The gold standard dose-to-mother stable isotope (deuterium oxide) technique [6] has significantly improved our understanding of human milk intake in recent years. This method does not interfere with normal feeding patterns and can accurately estimate the total volume of human milk consumed by an infant over two weeks (rather than over 24-h with test-weighing) – providing a markedly improved assessment of intake [7–9].

Recently, we published equations to predict human milk intakes based on data using the dose-to-mother stable isotope technique, and reported that mean (SD) human milk intake was 762 (257) mL/day in infants aged 7–10 months [10]. However, the distributions and correlates of human milk intake, and how these might differ from infant formula intake, were not examined. The objectives of this study were to: 1) estimate human milk intakes (by volume) in New Zealand (NZ) infants (7–10 months) using the dose-to-mother technique; 2) describe ‘total infant milk intakes’ (i.e. including infant formula) by age, and explore whether total infant milk intakes differ between infants who only consume human milk, those who only consume infant formula, and those who consume both; and 3) explore how infant, caregiver, household, and feeding characteristics correlate with human milk intakes and total infant milk intakes.

## Materials and methods

### Study design and participants

**Fig. 1:**
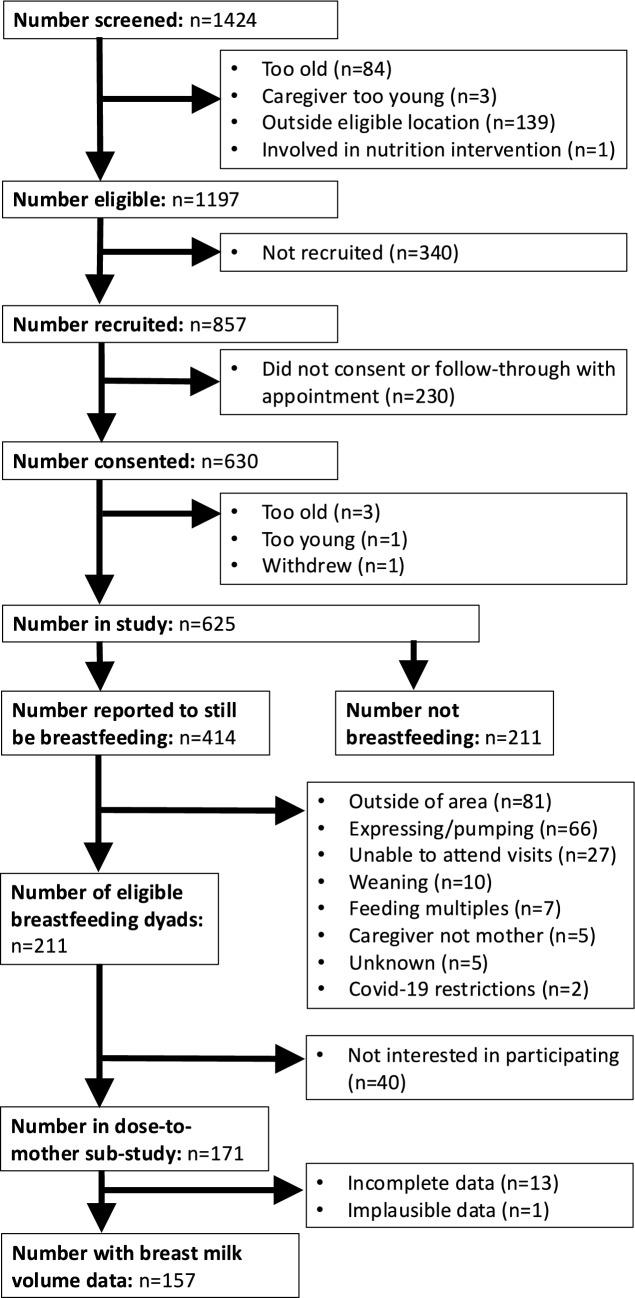
Flow chart of participant recruitment and available data

Data collection in this cross-sectional observational study occurred between July 2020 and February 2022, with detailed methods published elsewhere [11]. Within First Foods NZ (FFNZ), breastfeeding mother-infant dyads were recruited to participate in a dose-to-mother substudy (Fig. 1). Inclusion criteria were: breastfeeding, living in Dunedin or Auckland (urban centres in the North and South Islands of NZ), not having recently participated in a nutrition intervention study that might have changed infant feeding, ability to communicate in English, mother 16 years or older, and infant aged between 7.0–9.9 months. Participants were excluded if they expressed human milk as we would have been unable to control the intake of any previously expressed (non-deuterated) human milk which would not then be captured as part of the infant’s milk intake at the time of collection. Thus, the sample represented mothers who fed human milk from the breast only.

A sample size of 150 breastfeeding mother-infant dyads would enable an estimation of mean intake of ± 34 mL/day (i.e., 5% of expected total intake, [12]) at a 95% precision level. Ethical approval was obtained from the Health and Disability Ethics Committees New Zealand (19/STH/151) and all caregivers provided informed written consent.

### Sociodemographic characteristics and infant formula intake

Questionnaires were used to collect demographic information (caregiver: age, ethnicity, education, current employment status, parity, self-reported weight and height; household: area level deprivation, number living in the house, out-of-home childcare use; and infant: age, sex, ethnicity, and preterm status). For individuals identifying as more than one ethnic group, ethnicity was prioritised to one group using the following order: Māori, Pacific, Asian, Others, European [13].

Infant formula intake was determined by the average intake over two 24-h recalls using the multiple-pass method [14, 15]. This method included recording the brand of infant formula, amounts offered and consumed, along with the number of scoops and volume of water used to prepare the infant formula, which was used to calculate infant formula intake in FoodWorks 10 (Xyris Software, Australia). Caregivers reported whether the infant was unwell on the recall day. If “yes”, caregivers were asked whether the illness affected infant appetite (due to sickness, teething, colic, or feeling unwell).

### Anthropometry

Infant anthropometry was collected following the World Health Organization (WHO) protocol [16] and described in detail previously [17]. Age- and sex-specific body mass index (BMI) z-score was calculated using WHO Child Growth Standards [16]. For infants in the dose-to-mother subsample, weight and length were measured at both the first (day 0) and final (day 14) study visits, for the purpose of determining human milk intakes. Mothers in the dose-to-mother subsample had their weight (Tanita HD-351) measured in duplicate at the first (day 0) and final (day 14) study visits. Mother’s height was measured at the third study visit (day 7) using a portable stadiometer (Wedderburn Portable Height Rod WS-HRP). Mothers not in the dose-to-mother subsample self-reported weight and height from which BMI was calculated.

### Human milk volume assessment methods

The human milk intake of infants was assessed using the dose-to-mother stable isotope (deuterium oxide; D_2_O) technique, following a modified version of the International Atomic Energy Agency protocol [7] that used a shortened saliva collection protocol of four samples instead of the original design of seven. This method has been used safely in human studies for many years. Concentrations of D_2_O of less than 15% have not been associated with harmful effects in mammals [7], and 30 g D_2_O is approximately 0.08% of an average female adult’s total body water. A full detailed description of the dose-to-mother methodology and sampling technique are provided in Supplementary Methods. Briefly, baseline saliva samples were collected from both mother and infant. The mother then consumed an accurately measured dose of D_2_O (Aldrich, 99.8 atom % D). The elimination of D_2_O from the mother and the infant (measured through saliva) was measured over a 14-day period. Post-dose saliva samples were collected from both mother and infant at: day 2 (or day 3), day 8 (or days 7 or 9), and day 14 (or day 13). This reduced sampling protocol has been shown to have high sensitivity and specificity [9]. The deuterium enrichment (i.e. above natural abundance) in saliva samples was determined by Fourier-Transform Infrared Spectroscopy (Thermo Fisher Nicolet Summit, Thermo Fisher Scientific). Each sample was measured in duplicate, unless the difference between the two sample measurements was > 3 mg/kg, then a third or fourth measure was taken until two measures were < 3 mg/kg apart. The mean of two measurements (within 3 mg/kg) was the calculated D_2_O enrichment at each timepoint.

### Human milk intake calculation

The calculation of human milk intake of the infant was completed for each mother-infant dyad using the 3-day design Microsoft Excel template and manual by Liu and Duffull [18], which is based on the two-compartment steady-state model [7]. Briefly, intake of water from human milk and from sources other than human milk was calculated by fitting the outputs of D_2_O enrichment from both mother and infant to a model calculating water turnover. Non-linear regression was used to determine the value of the constants giving the best line of fit through the data points, after accounting for other inputs and losses [7, 8].

### Season

Participants were categorised into season of milk feeding based on the date of their first (day 0) visit. The NZ season classifications were: Summer: 1 December–28 February; Autumn: 1 March–31 May; Winter: 1 June-31 August; Spring: 1 September-30 November.

### Statistical analysis

Infant participants from the full FFNZ study who did not report any breastfeeds or consumption of human milk during their 24-h diet recalls were classified into a group that only consumed infant formula (“Infant formula only”). Participants from the dose-to-mother subsample were grouped into those who also consumed infant formula as measured in their 24-h diet recalls (“Mixed: human milk and infant formula”) and those who consumed only human milk and no infant formula (“Human milk only”). Participants in the wider FFNZ who were not part of the dose-to-mother subsample and reported having a breastfeeding occasion or human milk in their 24-h diet recalls were not included in this analysis (n = 291). Demographic characteristics were described for all samples and subgroups.

To describe human milk, infant formula, and total infant milk intakes, mean (SD) intakes were reported for all subsamples and subgroups. The distribution of human milk intakes for the dose-to-mother subsample were illustrated with histograms. Total milk intakes in the three subgroups and by age group were illustrated using box plots, with medians reported. The WHO suggests that a ‘low intake’ of milk is less than the mean − 2SD and a ‘high intake’ of milk is more than the mean + 2SD [19]. These reference cut-points were generated with the available data for each of the subgroups and by age group. The number and percentage of participants with low and high intakes was calculated.

Associations of demographic characteristics and feeding variables, with human milk and total infant milk intakes were estimated using regression models, adjusted for age. Mean differences and 95% CIs were calculated. No adjustment for multiple testing was undertaken, and p-values were not reported. Residuals of all models were plotted and visually assessed for homoskedasticity and normality. All statistical analysis was undertaken in Stata 18.0 (StataCorp, Texas).

## Results

The flow chart of participants through the FFNZ study is shown in Fig. 1. Of 211 eligible mother-infant dyads, 171 took part in the dose-to-mother subsample and 157 had complete human milk volume data. There were 211 FFNZ participants who were not breastfeeding and provided accurate infant formula intake data from the 24-h diet recalls. Six participants stated in the questionnaire that they were breastfeeding (non dose-to-mother), however, did not report any breastfeeding occasions during their 24-h diet recalls and were therefore reclassified into the infant formula only group (*n* = 217).

Table 1 shows the demographics of the full FFNZ sample (*n* = 625), dose-to-mother subsample (*n* = 157), and subgroups of interest. Mothers in the dose-to-mother subsample were less likely to use “out-of-home” childcare (8% vs 17%) and to be in full-time employment (5% vs 11%), and more likely to have an infant of European ethnicity (73% vs 55%) than those in the full FFNZ sample. The subgroup of infants who only consumed human milk had a higher mean BMI z-score (0.30 vs 0.18) compared to the full FFNZ sample. This group were also more likely to use childcare (24% vs 17%) and less likely to have a university education (54% vs 65%).

**Table 1:** Demographic characteristics of the full FFNZ sample, the dose-to-mother subsample, and those only consuming infant formula

		Full FFNZ sample	Dose-to-mother subsample			Infant formula only^a^
			Full subsample	Only human milk	Mixed (human milk and infant formula)	
n^b^		625	157	132	25	217
Infant characteristics						
Age, mean (SD) months		8.4 (0.8)	8.3 (0.8)	8.2 (0.8)	8.5 (0.8)	8.4 (0.9)
Sex, n (%) male		335	85 (54.1)	71 (53.8)	14 (56.0)	117 (53.9)
Ethnicity^c^, n (%)						
	Māori	131 (21.0)	25 (15.9)	19 (14.4)	6 (24.0)	57 (26.3)
	Pacific	44 (7.0)	3 (1.9)	3 (2.3)	0	20 (9.2)
	Asian	90 (14.4)	9 (5.7)	8 (6.1)	1 (4.0)	28 (12.9)
	Others	16 (2.6)	6 (3.8)	6 (4.6)	0	5 (2.3)
	European	344 (55.0)	114 (72.6)	96 (72.7)	18 (72.0)	107 (49.3)
BMI z-score^d^, mean (SD)		0.18 (1.00)	0.20 (0.96)	0.19 (0.97)	0.22 (0.92)	0.30 (1.04)
Preterm^e^, n (%)		46 (7.4)	11 (7.0)	11 (8.3)	0	23 (10.6)
Out-of-home childcare, n (%)		109 (17.4)	13 (8.3)	7 (5.3)	6 (24.0)	51 (23.5)
Caregiver characteristics						
Age, mean (SD) years		32.7 (4.9)	33.4 (4.5)	33.7 (4.3)	31.7 (5.2)	32.2 (5.3)
Relation to infant—mother, n (%)		617 (98.7)	157 (100)	132 (100	25 (100)	213 (98.2)
Education, n (%)						
	School	94 (15.0)	16 (10.2)	14 (10.6)	2 (8.0)	46 (21.2)
	Polytechnic or similar	125 (20.0)	39 (24.8)	30 (22.7)	9 (36.0)	54 (24.9)
	University	405 (64.8)	102 (65.0)	88 (66.7)	14 (56.0)	116 (53.5)
Current employment, n (%)						
	Fulltime	70 (11.2)	7 (4.5)	4 (3.0)	3 (12.0)	35 (16.1)
	Part-time	137 (21.9)	32 (20.4)	25 (18.9)	7 (28.0)	39 (18.0)
	Other^f^	418 (66.9)	118 (75.2)	103 (78.0)	15 (60.0)	143 (65.9)
Parity, n (%)						
	One	304 (48.6)	59 (37.6)	47 (35.6)	12 (48.0)	115 (53.0)
	Two	191 (30.6)	61 (38.9)	53 (40.2)	8 (32.0)	54 (24.9)
	Three	88 (14.1)	29 (18.5)	24 (18.2)	5 (20.0)	28 (12.9)
	Four or more	42 (6.7)	8 (5.1)	8 (6.1)	0	20 (9.2)
Ethnicity^c^, n (%)						
	Māori	85 (13.6)	13 (8.3)	11 (8.3)	2 (8.0)	37 (17.1)
	Pacific	32 (5.1)	3 (1.9)	2 (1.5)	1 (4.0)	15 (6.9)
	Asian	84 (13.4)	6 (3.8)	5 (3.8)	1 (4.0)	33 (15.2)
	Others	15 (2.4)	6 (3.8)	6 (4.6)	0	5 (2.3)
	European	409 (65.4)	129 (82.2)	108 (81.8)	21 (84.0)	127 (58.3)
BMI^g^, mean (SD) kg/m^2^		27.8 (6.4)	27.0 (5.8)	26.8 (5.9)	28.2 (5.2)	29.9 (7.4)
Household characteristics						
Area level deprivation^h^, n (%)						
	Deciles 1–3 (low)	180 (28.8)	57 (36.3)	49 (37.1)	8 (32.0)	56 (25.8)
	Deciles 4–7	282 (45.1)	66 (42.0)	52 (39.4)	14 (56.0)	92 (42.4)
	Deciles 8–10 (high)	163 (26.1)	34 (21.7)	31 (23.5)	3 (12.0)	69 (31.8)
Number in household, n (%)						
	2–3	251 (40.2)	52 (33.1)	43 (32.6)	9 (36.0)	90 (41.5)
	4–5	306 (49.0)	94 (59.9)	80 (60.6)	14 (56.0)	97 (44.7)
	6 or more	67 (10.7)	11 (7.0)	9 (6.8)	2 (8.0)	29 (13.4)

FFNZ: First Foods New Zealand study

^a^Participants were defined as consuming only infant formula if they consumed infant formula in their diet recalls and did not report any breastfeeds in their recalls

^b^The full dose-to-mother subsample of *n* = 157 participants was further split into *n* = 132 who only consumed human milk, and *n* = 25 who were mixed fed. Of the full FFNZ sample, *n* = 217 were only fed infant formula. The remaining participants in the full FFNZ sample were infants who were breastfed but were not part of the dose-to-mother subsample (*n* = 251)

^c^Multiple response ethnicity was prioritised to one ethnicity using the following order: Māori, Pacific, Asian, Others, European[13]

^d^BMI z-score determined using WHO Child Growth Standards [14]. Missing BMI z-score data were: *n* = 13 from the ‘full FFNZ’ sample’ *n* = 0 from the dose-to-mother subsample; and *n* = 5 from the ‘Infant formula only’ subgroup

^e^Preterm defined as born < 37 weeks gestation

^f^Not employed or on parental leave

^g^Caregiver BMI was calculated from self-reported weight and height. There was missing BMI data for 29 participants, 19 from the ‘Infant formula only’ subgroup. The dose-to-mother subsample did not have any missing BMI values

^h^Determined using the New Zealand Deprivation Index (NZDep) [29]. NZDep2018 combines 9 variables from the 2018 New Zealand national census to determine socioeconomic deprivation score for each Statistical Area 1 (a geographic area defined by Statistics New Zealand containing ~ 100–200 people, combining meshblocks). The deprivation score estimates the relative material and social deprivation for the area where the participant lives, where decile 1 represents areas with the least deprivation and decile 10 represents the area with the most deprivation

Infant milk intakes are shown in Table 2**.** The mean (SD) human milk intake in the dose-to-mother subsample was 785 g/day (264 g/day) or 762 mL/day (human milk density = 1.03 g/mL). This was higher (828 g/day) in those who consumed only human milk (no infant formula) and decreased with age. The mean standard error associated with the human milk volume estimates was ± 14 g/day, to a maximum of ± 27 g/day. Total infant milk intakes were around 820 g/day, regardless of the type of infant milk consumed (only human milk, only infant formula, or mixed), decreasing from around 900 g/day at 7- < 8 months to around 700 g/day at 9- < 10 months.

**Table 2:** Mean infant milk intakes^a^

		Dose-to-mother subsample^b^						Infant formula only^b^		*p *- value^c^
		Full sample		Human milk only		Mixed (human milk and infant formula)				
		n	Mean (SD)	n	Mean (SD)	n	Mean (SD)	n	Mean (SD)	
Human milk intake, g/day		157	785 (264)	132	828 (227)	25	556 (332)	217	0	
	7—< 8 months	60	862 (272)	52	911 (228)	8	547 (334)			
	8—< 9 months	69	790 (222)	60	800 (207)	9	719 (310)			
	9—< 10 months	28	605 (268)	20	694 (203)	8	383 (294)			
Infant formula intake, g/day		157	37 (114)	132	0	25	232 (193)	217	819 (222)	
	7—< 8 months	60	30 (110)			8	226 (226)	86	908 (216)	
	8—< 9 months	69	24 (85)			9	184 (169)	72	796 (177)	
	9—< 10 months	28	83 (166)			8	291 (194)	59	719 (232)	
Total infant milk intake, g/day		157	821 (237)	132	828 (227)	25	788 (289)	217	819 (222)	0.726
	7—< 8 months	60	892 (235)	52	911 (228)	8	773 (256)	86	908 (216)	0.247
	8—< 9 months	69	814 (233)	60	800 (207)	9	903 (368)	72	796 (177)	0.334
	9—< 10 months	28	688 (196)	20	694 (203)	8	674 (187)	59	719 (232)	0.816

^a^All infants were being fed solid foods in addition to their infant milk(s)

^b^Dose-to-mother subsample was a subsample of the First Foods New Zealand (FFNZ) study that was recruited from infants who were currently breastfed. The ‘infant formula only’ sample is a subgroup of FFNZ that reported infant formula intake in their diet recalls and did not report any breastfeeds in their diet recalls. Infant formula intake was determined by use of 24-h recall data

^c^*p*-value between ‘human milk only’, ‘mixed (human milk and infant formula)’, and ‘infant formula only’ subgroups for total infant milk intakes from a one-way ANOVA F-test

**Fig. 2:**
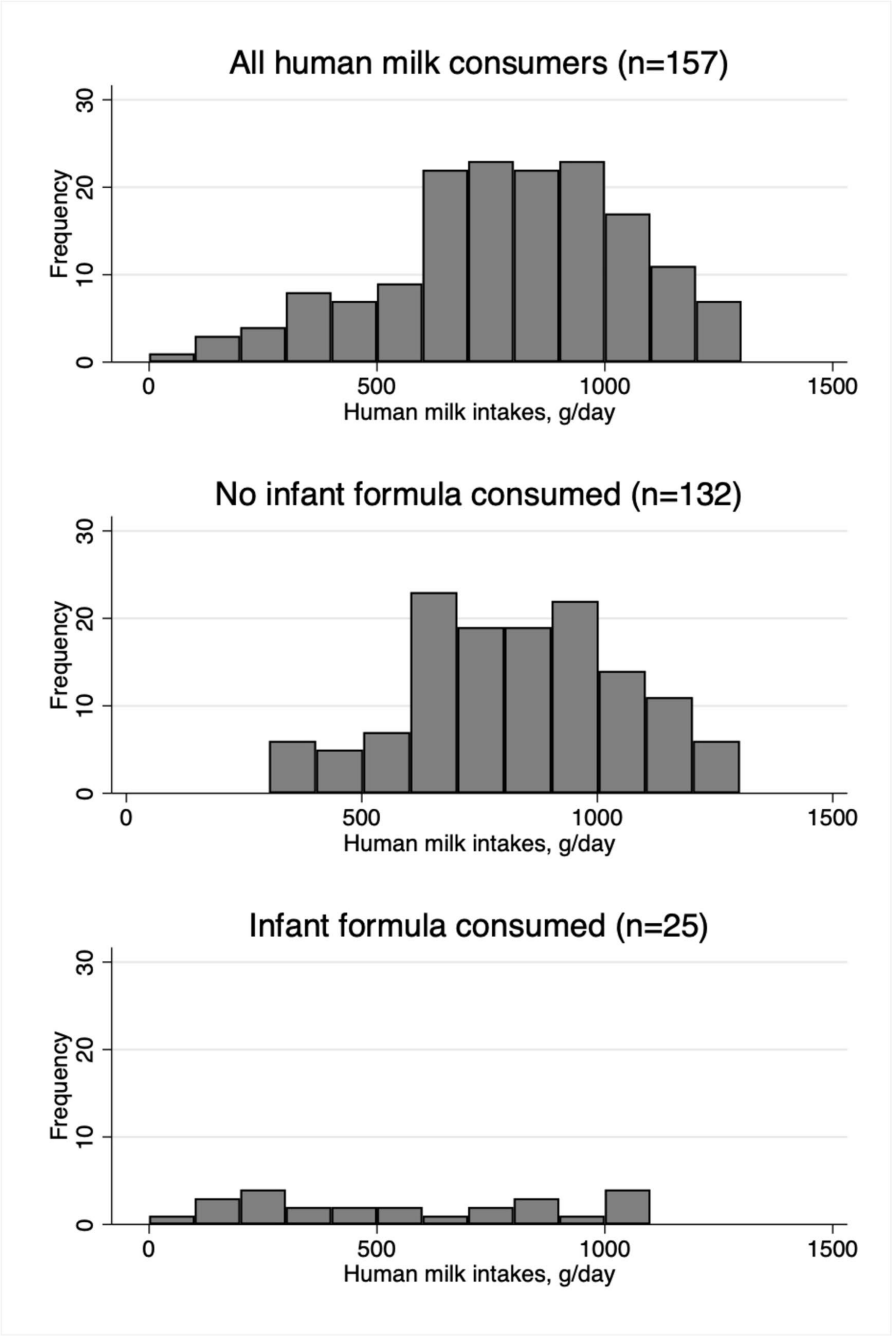
Histograms of human milk intakes in dose-to-mother subsample, by whether or not they also consumed infant formula

**Fig. 3:**
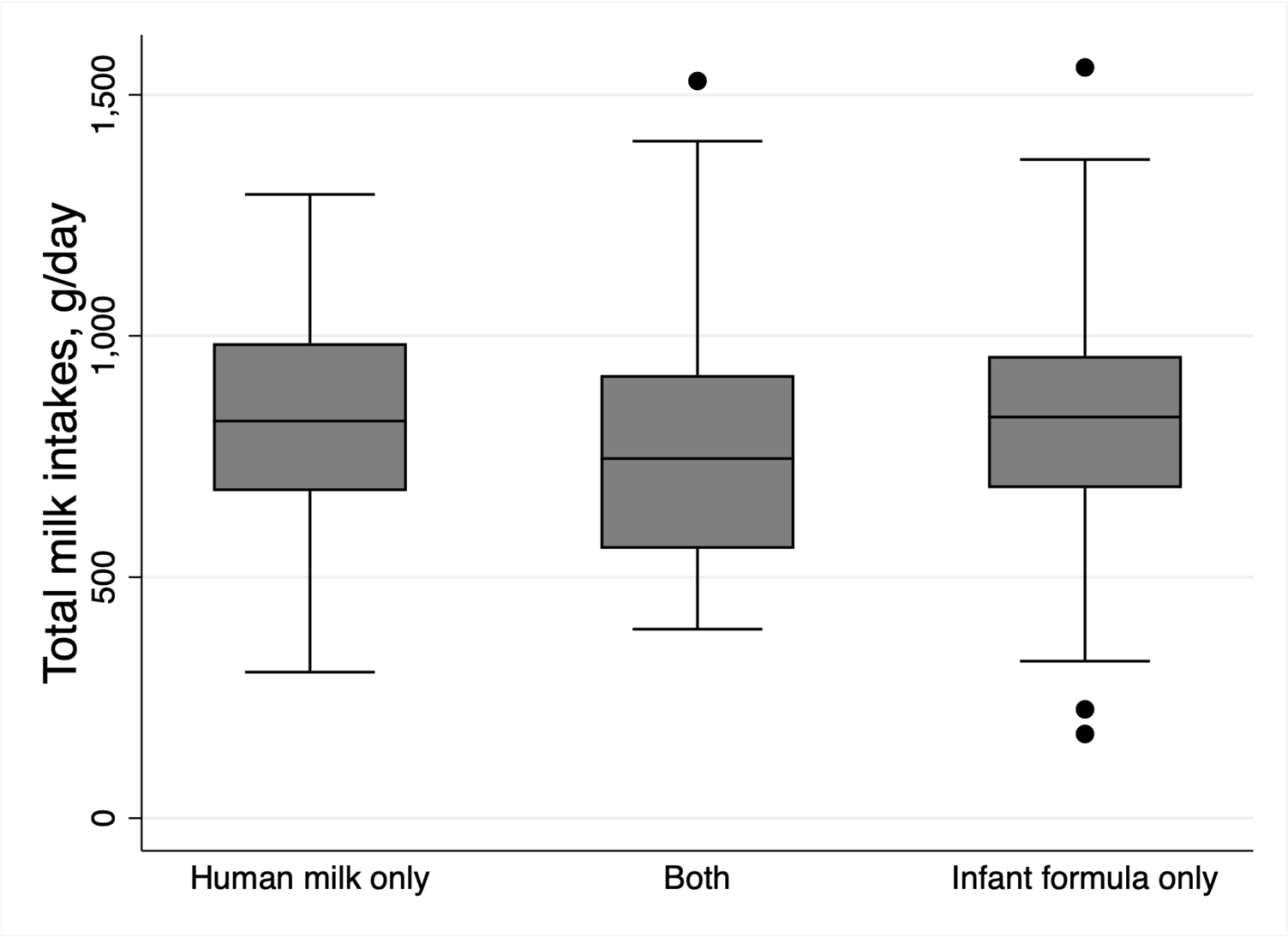
Box plots of total infant milk intake by whether infants consumed only human milk (*n* = 132); mixed: human milk and infant formula (*n* = 25); or only infant formula (*n* = 217)

**Fig. 4:**
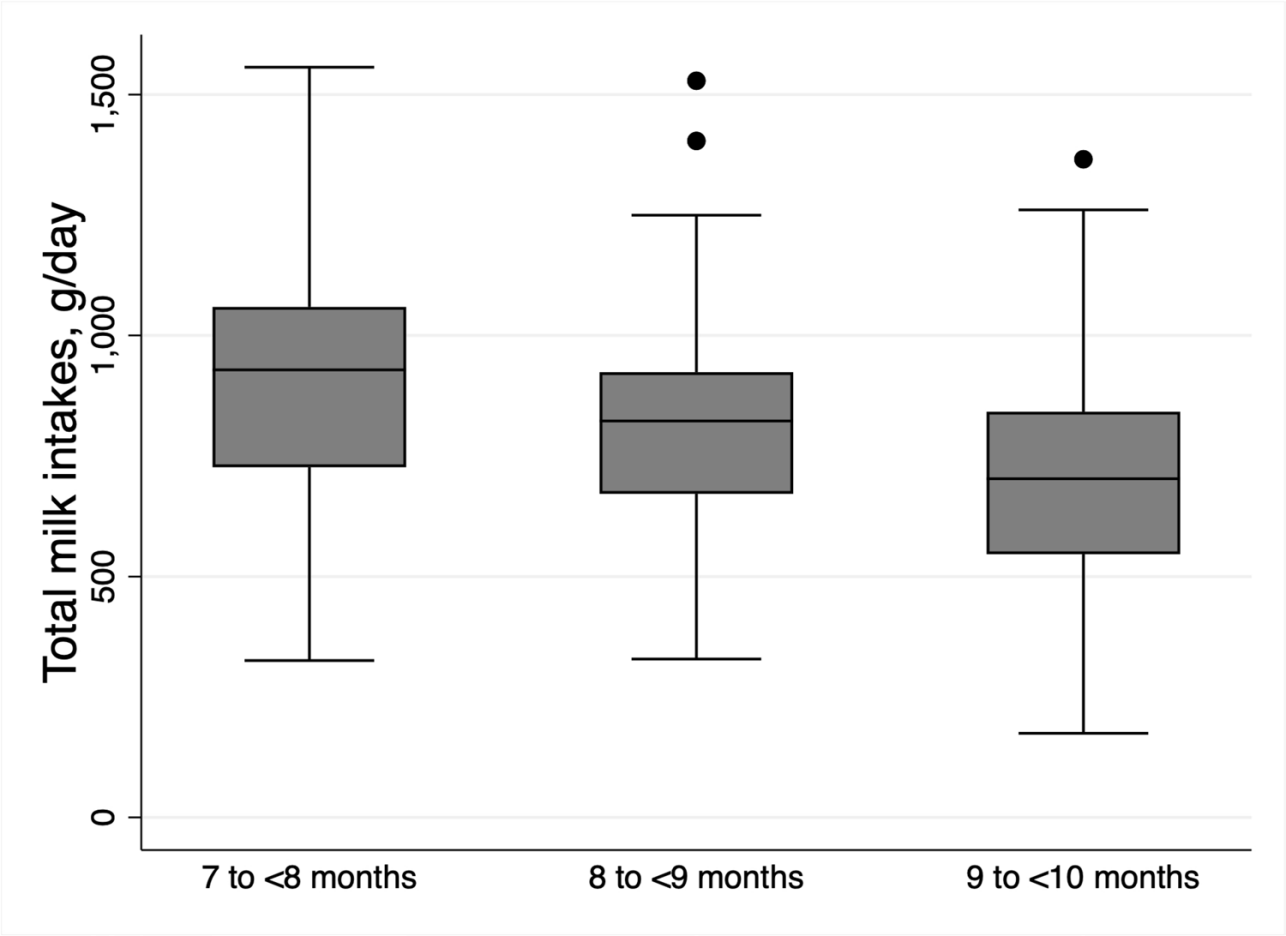
Box plots of total infant milk intakes by age: *n* = 146 for 7 to < 8 months; *n* = 141 for 8 to < 9 months; *n* = 87 for 9 to < 10 months

The distribution of human milk intakes (g/day) in the dose-to-mother subsample was normal with slight left-skew (Fig. 2), due to the consumption of infant formula by some infants (*n* = 25). Figure 3 illustrates the similarity in total infant milk intakes between infants who only received human milk (median 823 g/day) and infants who only received infant formula (median 831 g/day), while infants who received both had median intakes of 745 g/day, although this was not significantly different (median test *p* = 0.702). Box plots of total infant milk intake by age are shown in Fig. 4, showing decreasing total milk intake with increasing age. Histograms of total milk intake are in Supplementary Fig. 2. The median total milk intake in 7- < 8 month-old infants was 928 g/day, in 8- < 9 month-old infants was 822 g/day, and in 9- < 10 month-old infants was 702 g/day.

Supplementary Table 1 reports the low and high intake levels based on mean ± 2SD from the WHO [19], showing that a low intake of total infant milk was 346 g/day (from 455 g/day at 7- < 8 months to 268 g/day at 9- < 10 months) while a high intake was 1276 g/day (from 1348 g/day at 7- < 8 months to 1150 g/day at 9- < 10 months). Around 2% of the sample were considered low and 2% high consumers of total infant milk.

Table 3 describes the differences in human milk and total infant milk intakes by infant, caregiver, household, and feeding characteristics. Older infants had lower human milk and total infant milk intakes, while infants with a higher BMI z-score had higher human milk and total infant milk intakes. Male infants consumed, on average, 51 g/day more total infant milk than female infants. Mothers with lower education had infants with higher human milk intakes, while mothers who were working, either full-time or part-time, had infants with lower human milk intakes. Higher maternal BMI was associated with lower infant human milk intakes. There was no evidence of differences in infant milk intakes according to caregiver ethnicity, age, parity, household socioeconomic deprivation, or use of out-of-home childcare. Total infant milk intakes were lower if the infant was unwell and human milk intakes were lower if the infant was fed human milk on a schedule compared to being fed on demand. There was no evidence of differences in milk intakes by season (Supplementary Fig. 3).

**Table 3:** Infant, caregiver, household, and feeding characteristics as predictors of human milk intakes and total infant milk intakes

		Human milk intakes	Total infant milk intakes
		Mean difference (95% CI)^a^ g/day	
n		132	374
Infant characteristics			
Infant age, months		** − 109 (− 157, − 62)**	**− 96 (− 123, − 70)**
Infant sex			
	Female	Reference	Reference
	Male	41 (− 33, 115)	**51 (7, 94)**
Preterm birth			
	Term (≥ 37 weeks)	Reference	Reference
	Preterm (< 37 weeks)	− 81 (− 212, 50)	− 2 (− 77, 74)
BMI z-score^b^		**88 (54, 123)**	**59 (38, 80)**
Caregiver characteristics			
Age, years		− 2 (− 11, 6)	0 (− 4, 5)
	< 35 years	Reference	Reference
	≥ 35 years	− 26 (− 102, 49)	7 (− 40, 53)
Ethnicity^c^			
	Māori	73 (− 57, 203)	54 (− 11, 120)
	Pacific	− 135 (− 430, 160)	4 (− 99, 107)
	Asian	151 (− 37, 340)	7 (− 66, 79)
	Others	159 (− 16, 334)	40 (− 90, 170)
	European	Reference	Reference
Parity			
	Primiparous	19 (− 58, 97)	− 12(− 56, 32)
	Multiparous	Reference	Reference
Education			
	School	**143 (26, 261)**	0 (− 60, 60)
	Polytechnic or similar	64 (− 23, 151)	**69 (17, 121)**
	University	Reference	Reference
Current employment			
	Full-time	**− 268 (− 474, − 62)**	− 56 (− 126, 14)
	Part-time	**− 105 (− 196, − 15)**	− 41 (− 98, 15)
	Other	Reference	Reference
BMI^d^, kg/m^2^		**− 7 (− 13, − 1)**	− 1 (− 4, 2)
Household characteristics			
Out-of-home childcare			
	No	Reference	Reference
	Yes	− 59 (− 222, 105)	− 40 (− 98, 18)
Area level deprivation^e^			
	Deciles 1–3 (low)	Reference	Reference
	Deciles 4–7	61 (− 21, 143)	8 (− 44, 60)
	Deciles 8–10 (high)	− 36 (− 131, 59)	13 (− 45, 70)
Feeding variables			
Infant morbidity^f^			
	No	Reference	Reference
	Yes	− 26 (− 130, 78)	**− 70 (− 131, − 9)**
Demand or schedule feeding			
	Demand	Reference	
	Some schedule	**− 184 (− 266, − 103)**	
	Schedule	**− 217 (− 359, − 76)**	
Season^g^			
	Spring^h^	Reference	Reference
	Autumn	16 (− 92, 123)	− 8 (− 69, 53)
	Summer	− 33 (− 126, 59)	− 6 (− 64, 52)
	Winter	− 48 (− 152, 55)	− 37 (− 96, 21)

Bold indicates significant mean difference (*p* < 0.05)

^a^Mean differences (95% CI) determined using linear regression with all associations adjusted for infant age

^b^BMI z-score was determined using WHO Child Growth Standards [14]. No participants were missing BMI z-score data for ‘human milk intakes’, and five participants were missing BMI z-score data for ‘total infant milk intakes’

^c^Multiple response ethnicity was prioritised to one ethnicity using the following order: Māori, Pacific, Asian, Others, European[13]

^d^Caregiver BMI was calculated from self-reported weight and height. There was missing BMI data for 19 participants for ‘total infant milk intakes’. The dose-to-mother subsample did not have any missing BMI values

^e^Determined using the New Zealand Deprivation Index (NZDep) [29]. NZDep2018 combines 9 variables from the 2018 New Zealand national census to determine socioeconomic deprivation score for each Statistical Area 1 (a geographic area defined by Statistics New Zealand containing ~ 100–200 people, combining meshblocks). The deprivation score estimates the relative material and social deprivation for the area where the participant lives, where decile 1 represents areas with the least deprivation and decile 10 represents the area with the most deprivation

^f^Collected from 24-h diet recall data. Yes was if the infant was unwell and that the illness (including sickness, teething, colic or feeling unwell) affected their appetite the day of the 24-h recall. Nineteen participants (14.4%) were unwell when human milk intakes were assessed and 56 participants (18.7%) were unwell when total milk intakes were assessed

^g^Season of milk feeding was determined using the date of their first (day 0) visit

^h^Spring was chosen as the reference group as it represented the season with the largest group size (human milk intakes: spring *n* = 50, autumn *n* = 22, summer *n* = 35, winter *n* = 25; total milk intakes: spring *n* = 118, autumn *n* = 75, summer *n* = 89, winter *n* = 92)

## Discusssion

This study describes human milk and total milk intakes (including infant formula) in New Zealand infants aged 7–10 months, demonstrating wide variability in intakes. Our mean human milk intake (785 g/day) of older infants (7–10 months) is comparable to estimated intakes using dose-to-mother methods previously reported. Including older infants (6–9 months) from low to upper middle-income countries of around 750 g/day [20], younger 6-month-old Icelandic infants who also consumed complementary foods 818 g/day [21], 6–8.5 month old infants from Brazil (740 g/day) and The Gambia (755 g/day) [22], with lower intakes reported between 6–8.5 months in Bangladesh of 618 g/day [22]. Lower estimates were also reported in a recent systematic review using a meta-regression approach (713 mL/day, 694 mL/d, 671 mL/day and 647 mL/day at 7, 8, 9, and 10 months of age, respectively) [21]. That review included studies using test-weighing methods, which have been reported to measure significantly lower volumes than the dose-to-mother method [21].

Interestingly, our mean intakes in late infancy are similar to mean intakes of even exclusively breastfed younger infants (< 6 months of age) globally from dose-to-mother studies [4, 18]. In the current study, human milk intakes at 7- < 8 months (911 g/day) were higher than exclusively breastfed infants (2–5 months) in Indonesia of 811 g/day, however this fits with early suggestions that intakes of infants from lower-income countries are around 100 mL/day less than intakes of infants from Western countries [22].

Additionally, our results are in line with human milk intakes reported by the WHO using mostly older test-weighing studies from industrialised countries, where exclusively breastfed infants were consuming an average of 803 g/day between 6–8 months and 900 g/day between 9–11 months. In our study, infants who only consumed human milk were consuming an average of 828 g/day, falling between the values reported by the WHO [17]. The WHO reported that partially breastfed infants were consuming an average of 592 g/day between 6–8 months of age and 436 g/day between 9–11 months [17], which fits with our findings of 556 g/day of human milk on average in partially breastfed infants.

Interestingly, total infant milk intakes (including infant formula) were consistent across different feeding methods (human milk only, infant formula only, mixed fed), averaging around 820 g/day. Given the cross-sectional nature of the study, this reflects current feeding mode and intake, rather than describing feeding patterns since birth. It has been reported previously that average intakes of infant formula were higher than human milk in infants during the early postnatal period (to 6 weeks of age) [25], although this could be a result of breastfeeding technique still being established, or difficulties in accurately estimating intakes. Previous work has described differences in the growth patterns between milk feeding groups, with formula-fed infants typically being heavier during infancy [23, 24]. Therefore, while there appear to be differences in BMI z-scores between feeding groups in this study, these may be driven more by earlier feeding practices or variations in milk composition (not assessed herein) than current intakes.

The decline in total infant milk intake with age is as what would be expected when infants transition to a more diversified diet by increasing consumption of solid foods, and is consistent with declining intakes over infancy [18, 21]. In agreement with previous research [22], infants with higher BMI z-score had higher human milk intakes, although it is unclear whether these infants have greater energy needs because of a greater growth rate, or different feeding behaviours, leading to increased milk intakes and subsequent greater weight gain.

Maternal characteristics also played a role in infant human milk intakes. Mothers with lower education levels tended to have infants who consumed more human milk, possibly reflecting socioeconomic factors (e.g., employment and financial status) influencing breastfeeding practices. However, this finding is in contrast with a study in Sri Lanka, where higher education was associated with higher human milk intakes and was thought to be related to health knowledge [26]. In addition, maternal employment, particularly full-time work was associated with lower human milk intakes, possibly indicating the challenges working mothers face in sustaining breastfeeding. Maternal BMI was inversely associated with human milk intakes, as has been shown in another study [27], although not supported by a recent meta-regression analysis [21]. Infants who were breastfed on schedule consumed less human milk than those fed on demand, reflecting previous work which highlights the importance of lactation frequency for the maintenance of the supply–demand cycle [28].

This study has many strengths, including use of the gold standard dose-to-mother technique for measuring human milk intake, and the detailed reporting of infant intakes during the complementary feeding period. Limitations include the representativeness of the sample, which, although diverse, may not be generalisable to NZ as a whole, and to other countries. The small sample size of non-European ethnic groups in the dose-to-mother subsample may have limited the ability to draw conclusions about human milk intakes among infants of Māori, Pacific and Asian ethnicity. The study excluded mothers who expressed human milk, and therefore the current study results may not represent all breastfeeding dyads as feeding patterns, milk production and infant intake may differ between mothers expressing breast milk and those only directly fed at the breast.

Our study used a shortened four-sample dose-to-mother protocol rather than the standard seven, which reduces participant burden but may increase the influence of individual data-point error, although this approach has been validated with high accuracy. Finally, it is important to note that different calculation models (e.g., Liu & Duffull [16] and IAEA [7]) may yield differing estimates of human milk intake. However, to date, there is no consensus regarding the direction or magnitude of these differences.

In conclusion, this study provides valuable insights into human milk intakes and total infant milk intakes during late infancy in NZ. The use of accurate assessment methods enhances our understanding of infant milk feeding and highlights the importance of individualised approaches to promoting optimal nutrition and growth in infants.

## Supplementary Information

Below is the link to the electronic supplementary material.

Supplementary file1 (DOCX 319 KB)

## Data Availability

The data used and/or analysed in the present study are not publicly available due to ethical restrictions related to the consent provided by participants. An ethically compliant dataset may be made available by the corresponding author upon reasonable request and upon approval by the Health and Disability Ethics Committee/University of Otago Ethics Committee.
