# Supplementary material for: Human milk and total milk intakes of mixed fed infants: a cross-sectional study of infants aged 7–10 months
Source: Eur J Nutr. 2026 Apr 28;65(4):123. doi: 10.1007/s00394-026-03973-1 (PMC13124827; doi:10.1007/s00394-026-03973-1)
Supplement: Supplementary file 1 — Supplementary file1 (DOCX 319 KB) [file 394_2026_3973_MOESM1_ESM.docx]

**Supplementary Material**

**Supplementary Methods**. Dose-to-mother methodology

**Supplementary Figure 1.** Calibration curve of D_2_O enrichment and correlation between actual (gravimetrically measured) and calculated (spectra absorbance) enrichment

**Supplementary Figure 2.** Histograms of total infant milk intakes by age: n=157 consumed human milk (n=25 of these also consumed infant formula) and n=217 consumed infant formula only

**Supplementary Figure 3.** Box plots of human milk intake only (A) and total milk intake (B) by season

**Supplementary Table 1.** Low and high infant milk intakes

**Supplementary Methods** – Dose-to-mother methodology

**Human milk volume assessment methods**

Baseline saliva samples were collected from both mother and baby on day 0 (baseline) during the first visit (either in the home or at the research clinic). Participants were asked to ensure that they themselves, and their baby, had not been fed any food or drink for 30 minutes prior to saliva sample collection. This was to ensure that the sample would not be contaminated with any food or drink residue that may have affected the analysis. Mothers were checked for any open sores in their mouth or bleeding gums prior to sample collection. After baseline saliva samples were collected, the mother consumed an accurately measured dose of D_2_O (Aldrich, 99.8 atom % D) and the time when the dose was consumed was recorded. The dose amount was 30 g measured to the nearest 2 decimal places (doses ranged from a minimum of 30.00 g to a maximum of 30.09 g). To ensure the full dose was consumed by the participant the dose bottle was filled with 50 mL water, inverted, and consumed by the mother through a straw. This was repeated for a second time. D_2_O spreads evenly through the mother’s body water pool within a few hours and is transferred to the infant through breastfeeding. The elimination of D_2_O from the mother and the infant (measured through saliva) is tracked over a 14-day period (1). Saliva samples were collected from both mother and baby on 3 occasions after the dose had been administered on day 0, they were: day 2 (or day 3), day 8 (or days 7 or 9), and day 14 (or day 13). This reduced sampling protocol has been shown to have a high sensitivity and specificity (2).

Saliva sampling technique

Researcher and participant personal safety and hygiene were protected during sample collection by appropriate use of face masks, sanitiser and disposable gloves. The researcher placed two dental cotton rolls (Henry Schein premium dental cotton rolls) into the mother’s mouth to soak up saliva for 2 minutes, without the cotton rolls being touched by the mother’s hands. Once sodden, they were placed into a 10 mL syringe which was squeezed into a labelled tube (1.5 mL, screw top) until at least 1 mL saliva was extracted. A second tube was filled as a spare sample. Saliva samples were collected in the same way for the baby, although the researcher held one cotton roll and collected saliva by moving the cotton roll around the left and right sides under the baby’s tongue until sodden. Samples were placed in labelled plastic zip lock bags (mother and baby samples bagged separately) and into a cooler box with an ice pack (free from any D_2_O doses), and then transferred to a -20degC freezer, until analysis.

Saliva sample analysis of D_2_O by Fourier-Transform Infrared Spectroscopy

The deuterium enrichment (above natural abundance) in saliva samples was determined by Fourier-Transform Infrared Spectroscopy (Thermo Fisher Nicolet Summit, Thermo Fisher Scientific) with a liquid accessory attachment: SPECAC Pearl and CaF_2_ 100µm pathlength top and bottom window plates. A standard curve was prepared following the methods described by the IAEA (1). Gravimetrically prepared standards of D_2_O (Aldrich, 99.8 atom % D) were used to achieve target enrichments of 0, 100, 200, 400, 600, 800, 1000 and 1500 mg/kg. These standards were created using local tap water with D_2_O added in order to obtain D_2_O enriched standards above natural abundance (1). Additionally, two validation standards were prepared with target enrichments of 300 mg/kg and 1300 mg/kg.

A quantification method for D_2_O was set up using these standards created in TQ Analyst (Thermo Scientific) software. The region fixed location height for the D_2_O peak was at 2,504 cm^-1^ (1). Accurate results depend on good baseline estimates (3), so the curve (plateau point at either side of the bottom of the peak) was carefully assessed and determined to be between 2,665 and 2415 cm^-1^, as also previously reported (4). The final calibration curve is shown in **Supplementary Figure 1**.


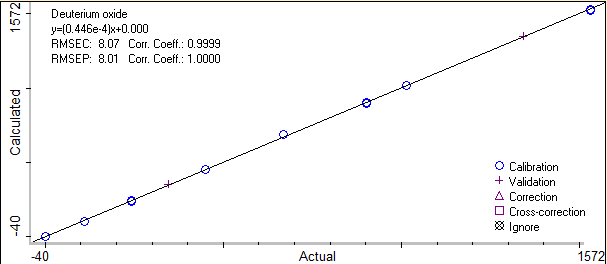


**Supplementary Figure 1.** Calibration curve of D_2_O enrichment and correlation between actual (gravimetrically measured) and calculated (spectra absorbance) enrichment

To assess the performance of the calibration curve, reference material of known D_2_O concentration was tested. The mean of triplicate measures of IAEA-604 (low) reference material was 139.84 mg/kg compared to the expected value of 138.00 mg/kg (Coefficient of Variation (CV) = 0.59%) and for IAEA-605 (medium) reference material was 1033.41 mg/kg compared to the expected value of 1037.00 mg/kg (CV = 0.19%). The measured enrichment should be as close to the reference value as possible, within 2%.

At the start of each week during the analysis period, the performance of the FTIR was checked by running maintenance checks on optimisation of the interferogram, laser calibration (measured frequency), and to check the machine was meeting specifications for noise, wavenumber and repeatability. To assess intra-day variability, daily analysis of the 1000 mg/kg standard was conducted (start and end of day), using a standard protocol. The calculated enrichment of the 1000 mg/kg standard was 1015.4 mg/kg. The mean analysed value was 991.6 mg/kg (SD = 8.0 mg/kg; CV = 0.80%). To assess day-to-day variability, a “spiked” saliva sample of known D_2_O volume was prepared and analysed daily (after the start of day calibration sample). This involved collecting approximately 30 mL of saliva and adding 0.00981 g D_2_O to approximately half of the saliva sample (17.502 g). The other half of the saliva sample was used as the background for analysing the spiked sample. The measured “spiked” sample mean was 603.5 mg/kg (SD = 5.3 mg/kg; CV = 0.88%).

A full day was required to analyse four mother-infant pair samples. Two sets of mother-infant pair samples were thawed at a time, at room temperature (30 minutes), inverted four times each and centrifuged at 1000 g for 10 minutes. Baseline mother or infant saliva samples were used as the background scan for each post-dose saliva sample. Exactly 45 µL of sample (background or post-dose) was pipetted onto the middle of the bottom cell window, then the top cell window was carefully placed onto the bottom cell window (avoiding air bubbles) and measured at the following settings:

Measaurement mode: Absorbance

Apodization: Triangular

Number of scans: 64

Resolution: 2.0

Range: min = 2300, max = 2,900

Analysed saliva were cleaned from the cell window using a delicate wipe (KIMTECH, Kimwipes) and 1 drop of pure ethanol to ensure the window plate was clean and dry before the next sample was analysed. Each sample was measured in duplicate, unless the difference between the two sample measurements was >3 mg/kg, then a third or fourth measure was taken until two measures were <3 mg/kg apart. The mean of two measurements (within 3 mg/kg) was the calculated D_2_O enrichment at each timepoint.


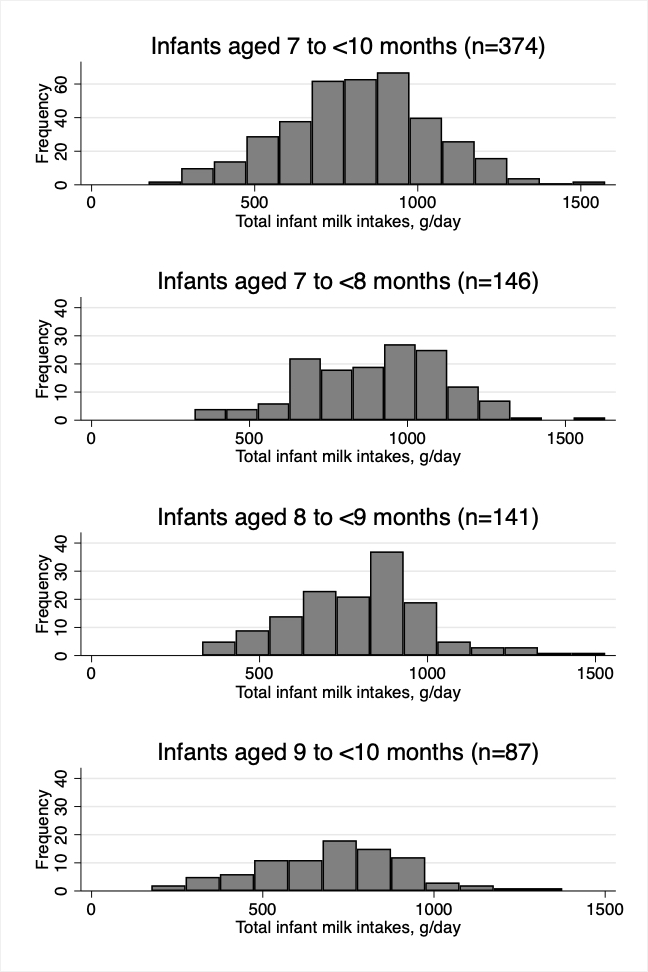


**Supplementary Figure 2.** Histograms of total infant milk intakes by age: n=157 consumed human milk (n=25 of these also consumed infant formula) and n=217 consumed only infant formula

**
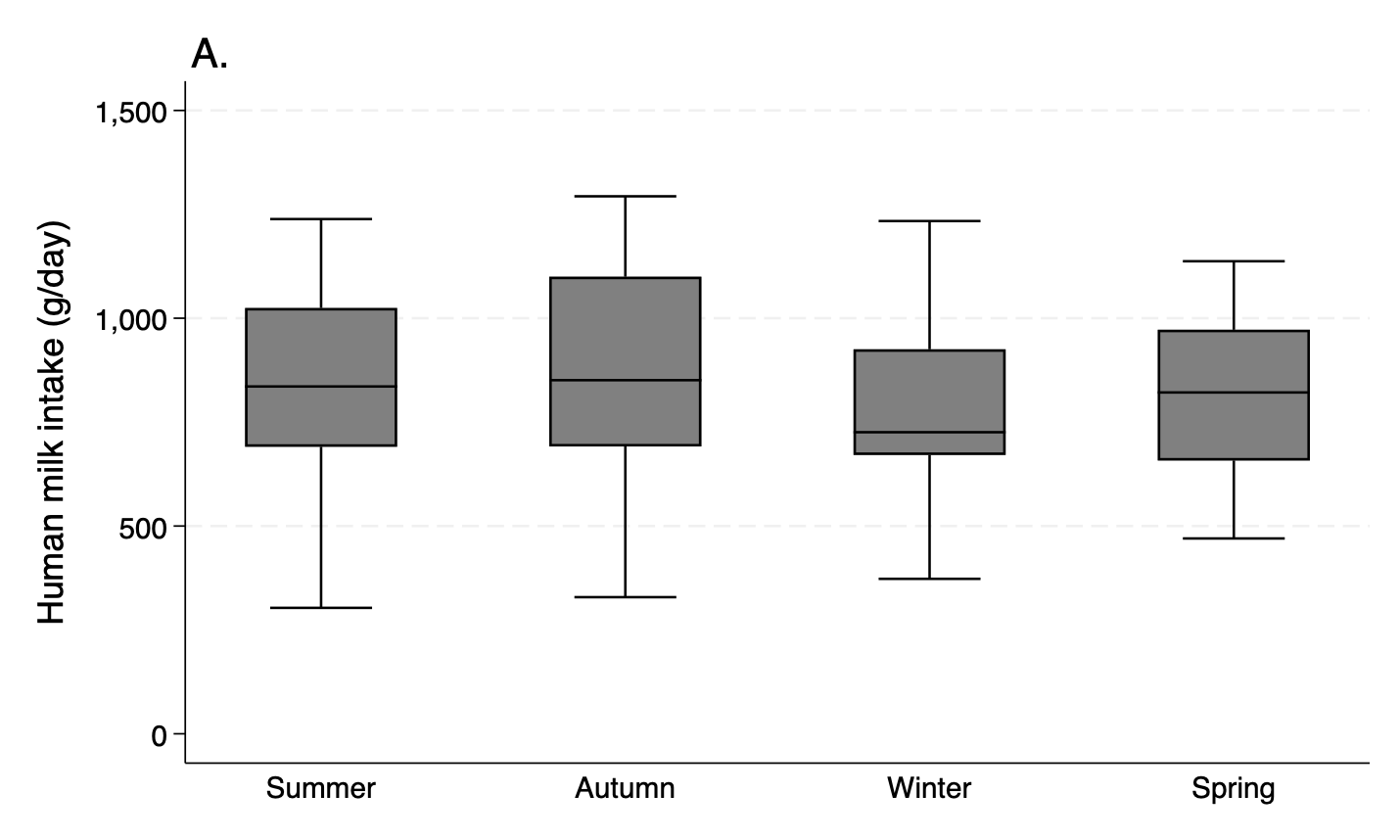
**

**
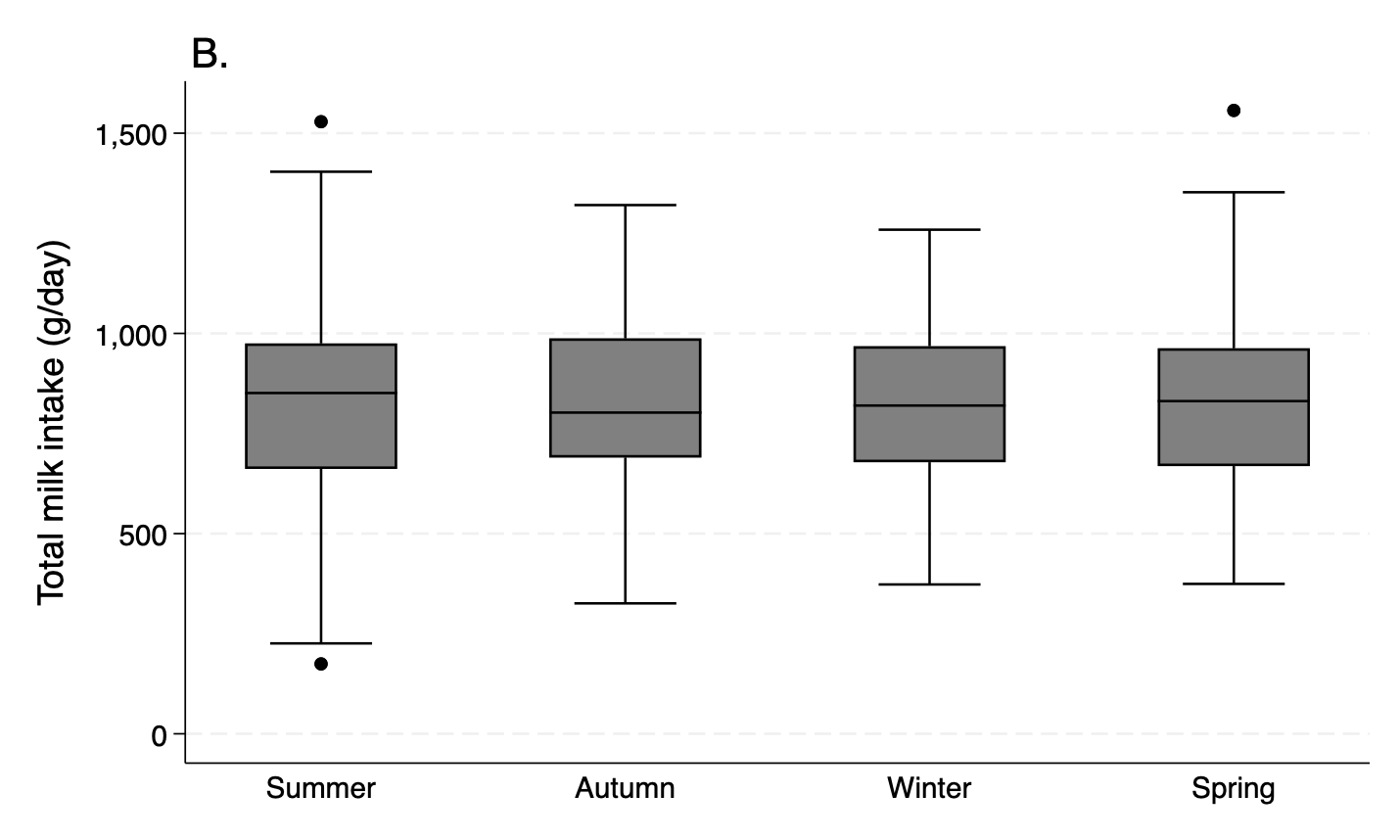
**

**Supplementary Figure 3.** Box plots of human milk intake only (A) and total milk intake (B) by season

**Supplementary Table 1.** Low and high infant milk intakes

|  | | **All** | **7 to <8 months** | **8 to <9 months** | **9 to <10 months** |
| --- | --- | --- | --- | --- | --- |
| n | | 374 | 146 | 141 | 87 |
| ‘Low intake’^a^ levels, g/day | |  |  |  |  |
|  | Human milk only | 375 | 454 | 387 | 287 |
|  | Infant formula only | 376 | 475 | 443 | 254 |
|  | Total infant milk | 346 | 455 | 394 | 268 |
| n (%) low consumers^b^ | | 6 (1.6) | 4 (2.7) | 3 (2.1) | 2 (2.3) |
| ‘High intake’^a^ levels, g/day | |  |  |  |  |
|  | Human milk only | 1281 | 1367 | 1214 | 1101 |
|  | Infant formula only | 1263 | 1341 | 1150 | 1184 |
|  | Total infant milk | 1276 | 1348 | 1216 | 1150 |
| n (%) high consumers^b^ | | 7 (1.9) | 2 (1.4) | 5 (3.6) | 2 (2.3) |

^a^ ‘Low intake’ defined as mean – 2SD; ‘high intake’ defined as mean + 2SD (5)

^b^ Low consumer defined as having less than the ‘low intake’ for total infant milk; high consumer defined as having more than the ‘high intake’ for total infant milk

**References**

1. International Atomic Energy Agency. Stable isotope technique to assess intake of human milk in breastfed infants. International Atomic Energy Agency; 2010 [cited 2025 Apr 14]. Available from: <https://www.iaea.org/publications/8168/stable-isotope-technique-to-assess-intake-of-human-milk-in-breastfed-infants>

2. Liu Z, Diana A, Slater C, Preston T, Gibson RS, Houghton L, Duffull SB. Development of a parsimonious design for optimal classification of exclusive breastfeeding. *CPT Pharmacometrics Syst Pharmacol* 2019;8:596–605.

3. Bluck L, Coward WA. Peak measurement in gas chromatographic mass spectrometric isotope studies. *J Mass Spectrom* 1997;32:1212–8.

4. Jennings G, Bluck L, Wright A, Elia M. The use of infrared spectrophotometry for measuring body water spaces. *Clin Chem* 1999;45:1077–81.

5. World Health Organization and UNICEF. Complementary feeding of young children in developing countries: a review of current scientific knowledge. 1998.
